# Complexity and Diversity of the Neurological Spectrum of SARS-CoV-2 over Three Waves of COVID-19

**DOI:** 10.3390/jcm13123477

**Published:** 2024-06-14

**Authors:** Justyna Jachman-Kapułka, Aleksander Zińczuk, Wojciech Szymański, Krzysztof Simon, Marta Rorat

**Affiliations:** 1 6th Department of Internal Medicine and Rheumatology, J. Gromkowski Specialist Regional Hospital, 51-149 Wroclaw, Poland; 2 1st Department of Infectious Diseases, J. Gromkowski Specialist Regional Hospital, 51-149 Wroclaw, Poland; alek.zinczuk@gmail.com (A.Z.); szymanski.wojciech9@gmail.com (W.S.); krzysimon@gmail.com (K.S.); 3 Clinical Department of Infectious Diseases and Hepatology, Wroclaw Medical University, 50-369 Wroclaw, Poland; 4 Department of Social Sciences and Infectious Diseases, Medical Faculty, Wroclaw University of Science and Technology, 50-370 Wroclaw, Poland; marta.rorat@pwr.edu.pl

**Keywords:** coronavirus, respiratory tract disease, consciousness disorder, genetic diversity

## Abstract

**Background/Objectives:** SARS-CoV-2 continually mutates, with five identified variants. Many neurological manifestations were observed during the COVID-19 pandemic, with differences between virus variants. The aim of this study is to assess the frequency and characteristics of neurological manifestations during COVID-19 in hospitalized patients over three waves in Poland with comparison and analysis correlation with the course of infection. **Methods:** This retrospective single-center study included 600 consecutive adults with confirmed COVID-19, hospitalized during 3 waves (pre-Delta, Delta and Omicron) in Poland. Demographic and clinical information and neurological manifestations were collected and compared across three periods. **Results:** The median age of the study group was 68, lower during the Delta wave. In the Omicron period, the disease severity at admission and inflammatory markers concentration were the lowest. Neurological manifestations were observed in 49%. The most common were altered mentation, headache, myalgia, mood disorder, ischemic stroke and encephalopathy. Smell and taste disturbances (STDs) were less frequent in the Omicron period. Neurological complications were predominant in the pre-Delta and Omicron periods. Ischemic stroke was observed more often in pre-Delta period. Altered mentation was related to higher severity at admission, worse lab test results, higher admission to ICU and mortality, while headache reduced mortality. Pre-existing dementia was related to higher mortality. **Conclusions:** Neurological manifestations of COVID-19 are frequent, with a lower rate of STDs in the Omicron period and more often cerebrovascular diseases in the pre-Delta period. Headache improves the course of COVID-19, while altered mentation, stroke and neurological comorbidities increase severity and mortality.

## 1. Introduction

Coronavirus disease 2019 (COVID-19) is an illness caused by severe acute respiratory syndrome coronavirus-2 (SARS-CoV-2) and it was first reported in December 2019 in the Chinese province of Wuhan [1]. Due to the high contagiousness and worldwide transmissibility of SARS-CoV-2, on 11 March 2020 the World Health Organization (WHO) declared COVID-19 as a pandemic with serious health concerns [2,3]. On 5 May 2023, the WHO Director-General published a statement that COVID-19 is now an established and ongoing health issue and no longer constitutes a public health emergency of international concern (PHEIC) [4]. After more than three years since the beginning of the first infection wave, over 773 million cases of COVID-19 and more than 7 million deaths from the disease were confirmed until the end of December 2023, according to the WHO COVID-19 dashboard.

Initially, COVID-19 was caused by a wild type of the virus, and afterwards SARS-CoV-2 began a continual evolution, which has persisted to the present [5]. The occurrence of mutations is the result of natural processes of adapting to the human body, rather than human intervention [6]. Since the onset of pandemic, multiple variants of SARS-CoV-2 have been identified and classified by scientific organizations and the WHO. A total of five variants of concern (VOCs) have been identified so far: Alpha, Beta, Gamma, Delta, and Omicron, causing subsequent waves of COVID-19. The Omicron variant is currently dominant globally. It has continued to evolve genetically and antigenically with an expanding range of sublineages (BA.1, BA.2, BA.4/BA.5) [7]. Many studies have revealed that different SARS-CoV-2 variants have variable features, including transmissibility, symptoms, hospitalization rate, mortality, severity and immune evasion [6,8,9,10]. The most severe course and the highest mortality rate were observed during the period of Delta dominance [6,9,10]. Based on current observations, mutations of SARS-CoV-2 will be promoting an increase in contagiousness rather than severity [6]. Since 2021, changes in the dominant SARS-CoV-2 variant, the implementation of vaccination programs, and the shift of infection to younger ages have decreased the detection of SARS-CoV-2 infections. These same factors, along with improved patient management and care, have significantly reduced the severity and mortality of COVID-19 [11].

Although SARS-CoV-2 mainly attacks the respiratory system, more than one-third of patients with COVID-19 exhibit concurrent neurological manifestations [12,13]. Multiple mechanisms are responsible for neurological consequences of the illness, most of which are indirect, including hypoxia, systemic illness, hypercoagulability, endothelial dysfunction of blood vessels in the central nervous system (CNS), general critical illness and inflammatory response [14]. Furthermore, SARS-CoV-2 triggers autoimmunity (by molecular mimicry, with activation of the innate immunity or prolong innate immunity), which develops extra-pulmonary COVID-19 diseases especially in younger population, for example autoimmune encephalitis, myopathy and inflammatory cardiac disease [15,16].

Nevertheless, SARS-CoV-2 has neurotropic and neuroinvasive properties, proven mainly on animal models [14]. In neural cells and olfactory mucosa there is an expression of angiotensin-converting enzyme 2 (ACE2), the receptor used for adsorption of the virus during SARS-CoV-2 infection [17,18]. Studies have identified several routes for SARS-CoV-2 entry into the CNS, mainly through the olfactory nerve terminals in the nasal cavity, leading to viral RNA presence in the olfactory bulbs and brain regions [19]. Via retrograde axonal transport, other cranial nerves, such as the vagus, glossopharyngeal, and trigeminal nerves, may also be potential paths for the virus to enter the brain [18]. Additionally, SARS-CoV-2 can spread through the circulatory system and penetrate the blood–brain barrier (BBB). The virus may also infect immune system cells, which can then transport SARS-CoV-2 through the BBB. Furthermore, the virus might propagate within the CNS through synapses and spread from one region of the brain to another [19]. Recent studies reveal that SARS-CoV-2 variants might also differ in their neuropathogenicity, and thus may lead to different neurological symptoms [20]. SARS-CoV-2 affects the normal physiological functions of the BBB and its cellular components and, as a consequence, contributes to the wide spectrum of clinically observed neurological manifestations. All the variants of the virus induce stress in CNS cells; however, the wild-type virus was cytopathic to all cell types except astrocytes, while Alpha and Beta variants were only cytopathic for pericytes, and the Omicron variant cytopathic for endothelial cells and pericytes. The wild-type virus increases BBB permeability and all variants, except Beta, modulate extracellular glutamate concentration, which can lead to excitotoxicity or altered neurotransmission [21].

Neurological symptoms may appear during COVID-19 infection at all stages (acute infection, subacute/post-infection, chronic phase) and may affect the CNS, peripheral nervous system (PNS), and muscles [13,22]. Manifestations of CNS involvement are reported in up to 25% of COVID-19 cases commonly with non-specific symptoms, including dizziness, headache, fatigue and altered mentation; less often, meningoencephalitis, cerebrovascular events, seizures and CNS neuro-immunological disorders [13,23]. PNS involvement has been reported in about 10% of COVID-19 patients, with smell and taste impairment the most common; and rarely, polyneuropathy, Guillain–Barré syndrome (GBS), and Miller Fisher syndrome [13,24,25]. Myalgia and skeletal muscle damage, with rare cases of rhabdomyolysis, have also been reported in the course of COVID-19 [22]. Some studies have shown differences in COVID-19 neurological manifestation among the virus variants, mainly in children population, where more severe neurological complications of COVID-19 were observed in Omicron wave [26,27,28]. Among adults, only a few studies, from other demographics, were conducted, revealing different percent distribution of neurological manifestations across the pandemic, and hence possible different courses of COVID-19 [27,28].

The study’s main objective is to assess the frequency, characteristics and differences of neurological symptoms and complications in the course of COVID-19 in hospitalized patients across three waves (pred-Delta, Delta, Omicron) in Poland, but also to analyze the correlation with infection severity. To our knowledge, this is the first study on the Polish population addressing the comparison between three waves.

## 2. Materials and Methods

### 2.1. Study Design

This retrospective single-center study included adult (≥18 years of age) patients with COVID-19, hospitalized in J. Gromkowski Specialist Regional Hospital in Wroclaw (Poland), which was from the beginning of the pandemic transformed into a single-name COVID-19 hospital. Only cases with confirmed SARS-CoV-2 infection, based on positive results of a real-time reverse transcriptase–polymerase chain reaction (RT-PCR) or rapid antigen test, were included. Based on the dominant SARS-CoV-2 virus sequences in Poland as analyzed by GISAID (Global Initiative on Sharing All Influenza Data), the pandemic was divided into three periods/waves: pre-Delta (1 March 2020–30 June 2021), Delta (1 July–31 December 2021) and Omicron (1 January–30 June 2022) [9]. In each wave we took the first 200 consecutive, symptomatic patients, 600 in total. The patient selection method arises from several factors: (1) significant disparities in numbers of hospitalized patients across the three waves; (2) the assumption of analyzing cases from the onset of each wave, predating virus mutations, which was particularly noticed in Omicron variant; (3) hospital organization: contingent upon bed availability and staff experience—at the beginning of each wave, only the best-trained team cared for COVID-19 patients; (4) shifting priorities in hospital admissions during the later phase of the Omicron wave.

The physicians were responsible for the whole diagnostics and treatment of studied patients, including identification of neurological complications. All patient data were collected retrospectively from electronic medical record databases and anonymized. The following base demographic and clinical information was collected: age, sex, comorbidities (cardiovascular, neurological, respiratory and chronic kidney diseases, diabetes, neoplasm and obesity), COVID-19-related symptoms, including duration before admission, duration of hospitalization, oxygen peripheral blood saturation at admission (SpO2), SARS-CoV-2 vaccination status, respiratory support, treatment, bacterial coinfections, intensive care unit (ICU) admission and death. The severity of illness at admission was assessed by the 9-point WHO ordinal clinical severity scale [29]. The scores were defined as follows: (0) no clinical or virological evidence of infection; (1) ambulatory, no activity limitation; (2) ambulatory, activity limitation; (3) hospitalized, no oxygen therapy; (4) hospitalized, oxygen mask or nasal prongs; (5) hospitalized, non-invasive mechanical ventilation (NIMV) or high-flow nasal cannula (HFNC); (6) hospitalized, intubation and invasive mechanical ventilation (IMV); (7) hospitalized, IMV + additional support such as pressors or extracardiac membranous oxygenation (ECMO); (8) death. Additionally, laboratory results, including white blood count (WBC), hemoglobulin (HGB), platelet count (PLT), concentration of C-reactive protein (CRP), procalcitonin (PCT), interleukine-6 (Il-6), D-dimer, ferritin and lactate dehydrogenase (LDH) were collected.

Among the studied patients, all registered neurological manifestations were divided into neurological symptoms, which were mainly self-reported (headache, dizziness, myalgia, smell disorder, taste disorder, vision disorder, altered mentation, mood disorder, sleep disorder, paresthesia, paresis) and neurological complications, which were diagnosed according to medical data (cerebrovascular diseases like transient ischemic attack [TIA], ischemic stroke, hemorrhagic stroke and venous thrombosis, encephalopathy, seizure, ataxia, myoclonus, mononeuropathy, polyneuropathy, GBS, meningitidis, encephalitis). Additionally, the impact of neurological manifestations and neurological chronic diseases on the course of COVID-19 was analyzed. Finally, we compared all data across the three waves.

### 2.2. Statistics

The obtained results were subjected to statistical analysis. Descriptive statistics were presented using means, standard deviations, medians and quartiles for quantitative variables and counts with percentages for qualitative variables. The correlation between qualitative variables was assessed using the Chi-square test. The normality of the distribution of variables in the study groups was checked using the Shapiro–Wilk normality test. To examine differences between groups the Kruskal–Wallis test was used, because none of the analyzed quantitative variables had a normal distribution. The analysis was performed in Statistica 9.1 (StatSoft, Krakow, Poland). The *p* value < 0.05 was selected as the threshold of significance.

## 3. Results

### 3.1. Main Characteristics of the Patients

**Figure 1:**
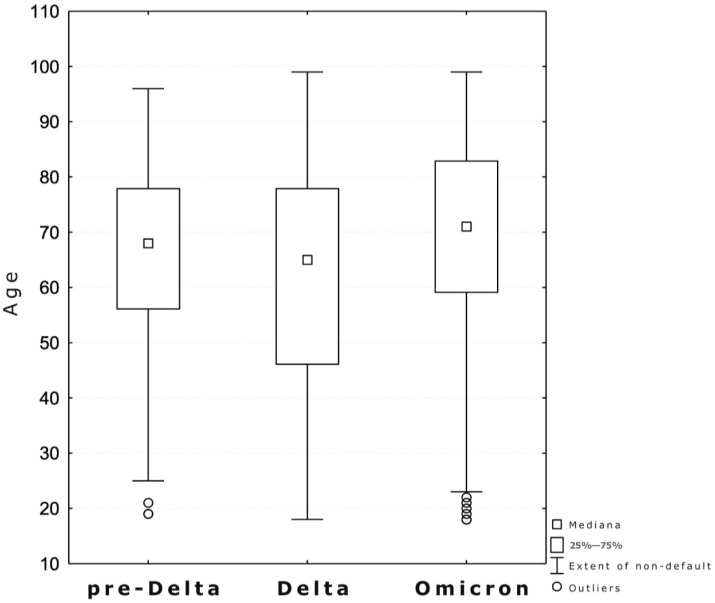
Age of patients (years) across three waves of COVID-19.

**Figure 2:**
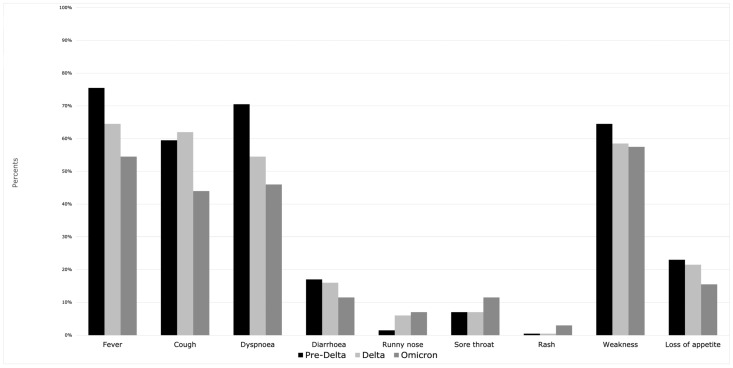
Symptoms of SARS-CoV-2 infection during three waves of the pandemic.

The study included 600 hospitalized patients, aged 18–99 (median 68 years), significantly younger during the Delta period than Omicron [65 vs. 71 years (*p* = 0.002)] (Figure 1). Half of the patients were male (*n* = 301), with similar distribution among the three periods (*p* = 0.917). The comorbidities (cardiovascular, respiratory diseases, dementia and cancer history) were statistically significant more often in the Omicron period. Furthermore, the largest number of patients vaccinated against SARS-CoV-2 was in the Omicron group. Duration of symptoms before admission was the shortest in the Omicron period (median 4 days, *p* < 0.001) and length of hospitalization was longer in the pre-Delta vs. Omicron wave (median 11.5 vs. 8 days, *p* < 0.001). Severity of COVID-19 at admission according to the WHO scale was the lowest in the Omicron period (*p* = 0.002). Among typical symptoms of SARS-CoV-2 infection (Figure 2), fever and dyspnoea occurred most frequently in the pre-Delta period. In the Delta period, cough appeared most often (*p* < 0.001), while runny nose was most common in the Omicron period (*p* = 0.025). Cases of severe respiratory failure requiring the use of high flow nasal oxygen therapy (HFNOT) or NIMV occurred most frequently in the Delta period (*p* < 0.001). Dexamethasone and tocilizumab were predominantly used in the Delta period (*p* < 0.001), whereas remdesivir (RDV) and molnupiravir (MPV) (*p* = 0.001, *p* < 0.001, respectively) in Omicron. Bacterial coinfections were the rarest in the Delta period (*p* < 0.001). There were no significant differences between pre-Delta, Delta and Omicron waves in the number of ICU admissions and deaths. All demographic and clinical characteristics of patients with COVID-19 are recorded in Table 1. Among analyzed laboratory parameters, the statistically significantly lowest levels of inflammatory markers were seen in the Omicron period (Table 2).

### 3.2. Neurological Manifestation

**Figure 3:**
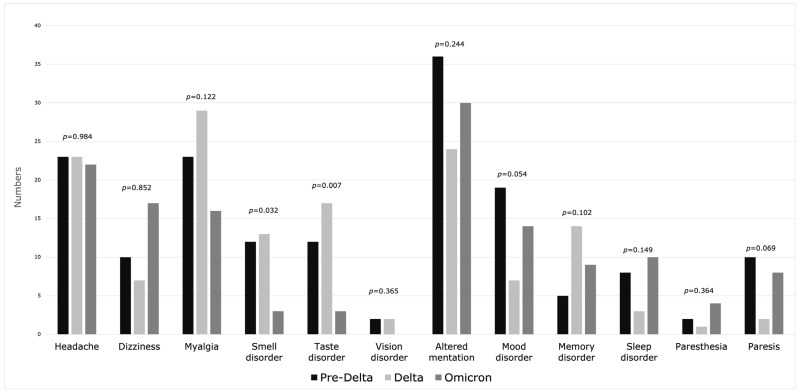
Neurological symptoms during three waves of COVID-19.

**Figure 4:**
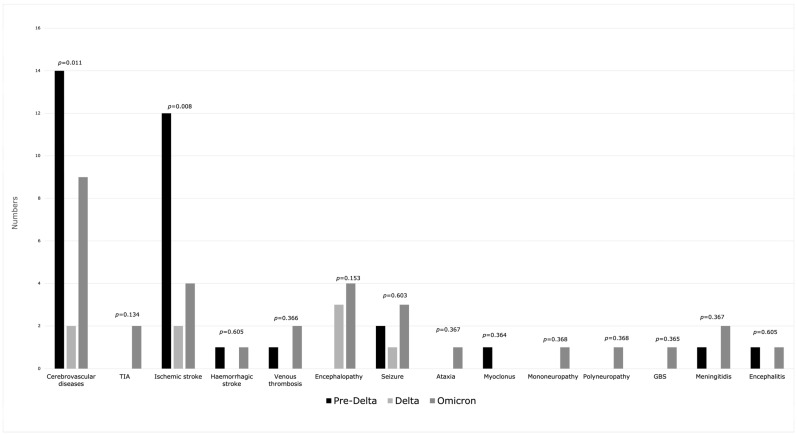
Neurological complications during three waves of COVID-19.

General neurological manifestations in the course of COVID-19 were observed in half of the patients (*n* = 294), with no statistical difference between the three waves: pre-Delta 49.5% (*n* = 99), Delta 50.5% (*n* = 101), Omicron 47% (*n* = 94) (*p* = 0.771). The most common symptoms (Figure 3), without statistical differences between periods, were altered mentation (15% of all), headache (11.33% of all), myalgia (11.33% of all) and mood disorder (6.67% of all). Smell and taste disorders were reported significantly less often in the Omicron period (smell *p* = 0.032, taste *p* = 0.007). Neurological complications (Figure 4) were observed in 6.83% (*n* = 41), with predominance in the pre-Delta and Omicron periods (*p* = 0.031). Ischemic stroke (3%, *n* = 18) and encephalopathy (1.17%, *n* = 7) were the most frequent. Although cerebrovascular diseases and ischemic stroke were observed more often in the pre-Delta period (*p* = 0.011, *p* = 0.008, respectively), due to the small number of cases, it is difficult to conduct a comparative analysis. Table S1 (Supplementary Materials) contains a comparison of neurological manifestations.

The occurrence of any neurological manifestations was not dependent on sex. Altered mentation was recorded more often in elderly patients regardless of the period (*p* < 0.001), while headache and myalgia dominated in the younger patient group (*p* < 0.001 and *p* = 0.004, respectively). Altered mentation was associated with higher illness severity at admission (*p* < 0.001), hospitalization in ICU (*p* = 0.01) and mortality (*p* < 0.001). In our analysis, deaths were more frequent among patients with at least one neurological manifestation and with stroke during the Omicron period (*p* = 0.03). Deaths were more frequent when no headache was reported (*p* = 0.02). The percentage of ICU admission was higher among patients with neurological complications (*p* < 0.001).

Some comorbidities influenced the appearance of neurological manifestations. For example, the presence of altered mentation was significantly higher among patients with any kind of comorbidities and cardiovascular diseases (*p* < 0.001 and *p* = 0.046, respectively). Pre-existing dementia had the greatest impact on the occurrence of altered mentation in all periods (*p* < 0.001). Ischemic stroke was more frequent when there was a history of heart diseases, hypertension and atrial fibrillation (*p* = 0.036, *p* = 0.026 and *p =* 0.027, respectively). On the other hand, myalgia and headache occurred more often when there were no comorbidities, like cardiovascular diseases (*p* = 0.046 and *p* < 0.001, respectively).

The presence of altered mentation was associated with worse lab test results. Duration of hospitalization was longer in patients with any neurological manifestation in the pre-Delta period (*p* = 0.005) and in patients with at least one neurological complication in the Omicron period (*p* = 0.02). However, the presence of headache or myalgia was associated with shorter hospitalization in the Omicron period (*p* = 0.005, *p* = 0.02, respectively). Among the analyzed correlations between neurological comorbidities and the course of COVID-19, a history of dementia was related to a higher risk of death (*p* = 0.003), while duration of hospitalization was longer among patients with histories of TIA or stroke (*p* = 0.025).

## 4. Discussion

The study aimed to identify neurological manifestations during COVID-19 in hospitalized Polish population with emphasis differences between three periods of pandemic. In the research group, 49% of patients had at least one neurological manifestation, consistently across periods. The most common neurological manifestations were altered mentation (15%), followed by headache, myalgia, mood disorder, dizziness, smell, taste and memory disorder, and finally cerebrovascular disorder; with smell and taste disorder predominated in pre-Delta and Delta waves.

Neurological manifestations of COVID-19 were observed already at the beginning of the pandemic. In Wuhan, 36.4% of hospitalized patients displayed neurological manifestations [13]. Later in the literature, the frequency of neurological manifestations, regardless of the pandemic period, ranges from 18.5% to 82% [22,30,31,32], which is consistent with our results. In a Polish report issued by Wnuk et al. [33] analyzing the general occurrence of neurological symptoms during COVID-19 (study conducted between March and September 2020, including 200 consecutive hospitalized patients), 84.5% of patients experienced them, which is significantly more than in our research. The difference may result from a different methodology. The second study included data collected retrospectively and prospectively, thus the researchers were focused on medical histories. Additionally, symptoms such as fatigue, atrial hypotension and tachycardia were classified as neurological manifestations.

In our study neurological complications in general were more frequent in the pre-Delta and Omicron periods with predominance of smell and taste disturbances (STDs) in pre-Delta and Delta periods and tendency to higher rate of cerebrovascular diseases in the pre-Delta period. Our results are quite different from the literature on this subject. Some studies regarding a population of children emphasized an increase in severe neurological symptoms throughout the Omicron period, despite milder neurological signs during the pre-Omicron period [26]. Researchers mostly observed a significant rise in seizures (mostly simple febrile seizures) and alterations in consciousness during the Omicron period [26]. Moreover, some cases of children with severe neurological symptoms, like encephalopathy, were also identified [34].

There are only a few studies analyzing the differences in neurological symptoms across COVID-19 variants among adults. In one retrospective study from India comparing neurological manifestations in the first two waves of COVID-19 (June 2020 to January 2021 and April 2021 to May 2021) [27], headache, dizziness and acute cerebrovascular syndromes were more frequent in the first wave, and myalgia in the second wave [27]. In our study headache, dizziness and myalgia occurred with a similar percentage distribution among all waves; however, acute cerebrovascular diseases were also more frequent in the first wave. Different frequencies of neurological manifestation across the pandemic, than in our research, were shown in another study from India by Kulkarni et al. [28], comparing three waves of the COVID-19 pandemic. Encephalopathy was statistically more common in the first wave, peripheral neuropathy in the second wave and acute symptomatic seizures in the third wave. However, after comparison of individual neurological manifestations per 1000 COVID-19 admissions, encephalopathy, ischemic strokes and seizures were all more common in the Omicron wave [28]. On the other hand, a study from Romania [35] found anosmia and ageusia to be the most common in Delta period; similarly to our conclusion.

Smell and taste disorders were statistically significantly less frequent in the Omicron period (pre-Delta: 6% and 6% vs. Delta: 6.5% and 8.5% vs. Omicron: 1.5% and 1.5%). Similarly, in a study by Coelho et al., patients infected with more recent variants (Delta, Omicron) were at a significantly lower risk of developing associated chemosensory loss [36]. STDs consisting of anosmia/ageusia, hyposmia/hypogeusia and parosmia/parageusia are important PNS features of COVID-19, with an etiology highly suggestive of this, albeit with varied prevalence between 5% and 80% across countries and periods of the pandemic [19,30]. Although smell and taste abnormalities are observed three times more frequently in Caucasians than in Asian patients, in our group the prevalence of STDs was quite low (8.5%–1.5%) [37]. This discrepancy may be due to the retrospective nature of the study and the subjective character of the symptoms. Furthermore, the literature records an increased prevalence of STDs in younger patients with more benign courses of COVID-19 [38]. Our hospitalized patients were older, frequently with altered mentation and severe course of COVID-19, which may additionally impact our statistics.

Patients with cerebrovascular diseases, particularly ischemic stroke, were too few in the study’s cohort to conduct proper statistical analysis; however, there was a higher rate of stroke cases in the pre-Delta period (pre-Delta 6% vs. Delta 1% vs. Omicron 2%). This is similar to reports in the study by Sureshbabu et al. [27]. On the other hand, Briciu at al. reported the highest incidence of ischemic strokes (2.08%) in the Omicron wave, although coagulopathy occurred more often in the Delta wave [39].

The following research, in addition to the neurological manifestations of COVID-19, presents a general characteristic of SARS-CoV-2 infection across three waves (pre-Delta, Delta, Omicron). Our observations of overall differences between waves are similar to other comparative studies conducted in Poland [10,40], as well as globally [6,8,41,42,43]. There was no sex predominance in any period; however, some studies emphasize higher hospitalization rates among men [10,41]. Patients in the Delta period were the youngest, while in Omicron they were the oldest, which is in line with other reports [10,41,42,43]. Duration of symptoms before hospitalization was the shortest in the Omicron period, probably due to greater social awareness, easier access to health care with better health organization and the availability of rapid antigen tests. On the other hand, the duration of stay in hospital was the longest in the pre-Delta wave, which was due to the lack of knowledge about the time of infectiousness of SARS-CoV-2.

Among the three waves in our research, the Omicron period in particular revealed the greatest differences compared to other waves. The severity of COVID-19 at admission was the lowest. Vaccination coverage was the highest in the Omicron period, which resulted from the greater availability of vaccinations. The higher presence of comorbidities in the Omicron wave may have, hypothetically, resulted from the fact that the cohort was the oldest. The treatment applied for COVID-19 varied across individual waves of the pandemic, due to changing recommendations, availability and emergence of new drugs, as well as severity. In the Omicron period, patients needed oxygen therapy less frequently (due to less severe course), and treatment with RDV/MPV was the most common. It obviously resulted from the less severe course of the disease and greater availability of drugs, but also shorter duration of symptoms before admission and faster referral to hospital. In addition, laboratory test results were better at that wave. Taking the foregoing into account, it might be surprising that among the patients in the study, the percentage of ICU admissions and deaths did not differ during the waves. As various studies have shown, the Delta variant of SARS-CoV-2 has a shorter incubation period and shorter time of evolution towards critical forms of the disease, with higher probability of oxygen therapy, ICU hospitalization and death than other variants [41,44,45]. On the other hand, Omicron has the greatest transmissibility due to multiple mutations [41,46]. It replicates more efficiently in the human bronchus than in lung tissue, with greater preference for involvement of the upper respiratory tract [41,47]. Patients with Omicron variant are more asymptomatic or with milder symptoms and in general report better outcomes with lower risk of hospitalization, ICU admission and death [6,48,49]. Lower hospitalization and mortality rates among patients with the Omicron variant are also related to protection induced by COVID-19 vaccines and previous SARS-CoV-2 infections [41,50]. In our study, the rate of ICU admissions and deaths is comparable in all waves, probably due to the numerous admissions of patients with even mild symptoms for the purpose of isolation, especially during the first two waves. On the other hand, the cohort of patients with the Omicron variant was older, with many comorbidities which increased the risk of death, regardless of the severity of COVID-19. The percentage of causes of death other than COVID-19 was therefore obviously the highest in the Omicron period (*p* = 0.018), although the number of cases is small, which may impact the results.

From the beginning of COVID-19, prognostic and risk factors of the course and severity of COVID-19 were taken into account. In this study, the presence of any neurological manifestation was associated with a higher risk of death. Altered mentation was associated with higher severity of illness at admission, higher rate of ICU admissions and deaths. Also, ischemic stroke had an impact on higher mortality rates in the Omicron period. In a research conducted in Poland by Drabik et al., the presence of high-risk neurological symptoms or signs, including decreased level of consciousness and stroke/TIA, increased the risk of in-hospital mortality in SARS-CoV-2 infection 3.13- and 7.67-fold, respectively [51]. On the other hand, headache and myalgia, reported more often in the younger group, were associated with shorter hospitalization time; additionally, the presence of headache increased the chance of survival. The presence of headache as a protective factor against death due to COVID-19 was confirmed in our study and several others [33,51,52,53,54,55]. Headache during COVID-19 may be a marker of the host’s defensive responses to enhance survival [53]. Varied pathophysiological mechanisms might mediate this effect. The cytokine storm is associated with pulmonary inflammation and/or vasoconstriction mediated, among others, by interleukin 6 (Il-6). Il-6 levels were lower and more stable in COVID-19 among patients that reported headache [53].

Neurological chronic diseases also influence the course of COVID-19. In the present research, a history of dementia (including Alzheimer’s disease and dementia for any other reason) increased mortality; in turn, a history of TIA/stroke prolonged the hospitalization period in the Omicron wave. In the relevant literature, coexisting neurological diseases significantly contributed to the course of COVID-19, increasing the mortality rate by up to 30% [56,57,58]. Possible mechanisms include immunosenescence, heightened IFN responses or genetic predisposition to severe COVID-19 (OAS1, APOE ε4 allele) in Alzheimer’s disease and susceptibility to acute stress in cerebrovascular diseases [59].

This study has some limitations. Firstly, it is a retrospective study from one medical center, with a limited number of cases and medical data. Secondly, the lack of availability of additional tests in all cases, such as MRI or EEG during the pandemic, may have resulted in underestimation of neurological complications and misdiagnosis. Thirdly, due to the severe state of our patients, medical histories were not fully completed in some cases.

## 5. Conclusions

Our study highlights the incidence of neurological manifestations among patients with SARS-CoV-2 infection in Poland. The clinical spectrum of neurological manifestations during the three pandemic waves did not differ much, but a low rate of taste and smell disorders in the Omicron period and higher incidence of cerebrovascular diseases in the pre-Delta wave were observed. Headache entailed a good prognosis for the course of COVID-19. On the other hand, altered mentation and recent stroke during COVID-19, as well as neurological comorbidities like dementia or past stroke, increase the severity of the course and mortality. As SARS-CoV-2 will remain permanently present in the human population, further studies on the occurrence of neurological manifestations during COVID-19 should be carried out, especially considering the continual mutation of SARS-CoV-2.

## Figures and Tables

**Table 1:** Demographic and clinical characteristics of patients hospitalized with COVID-19 and analysis according to pandemic waves. Quantitative parameters and variables are presented as mean values (standard deviation—SD) and median [interquartile range—IQR], and qualitative variables are presented as frequency/number.

Variables	Total Patients (*n =* 600)	Pre-Delta (*n =* 200)	Delta (*n =* 200)	Omicron (*n =* 200)	Chi^2^ *p*-Value
Age (years)	65.29 (18.56) 68 [53–80]	65.66 (16.56) 68 [56–78]	62.14 (19.3) 65 [46–78]	68.07 (19.28) 71 [59–83]	0.002
Sex					
Male	301 (50.16%)	98 (49%)	101 (50.5%)	102 (51%)	0.917
Female	299 (49.83%)	102 (51%)	99 (49.5%)	98 (49%)	
Duration of symptoms before admission (days)	6.28 (4.82) 6 [3–8]	6.88 (5.33) 7 [3–10]	6.55 (4.03) 7 [3–8]	5.34 (4.88) 4 [2–7]	<0.001
Length of hospitalization (days)	11.62 (9.01) 9 [6–14.5]	13.94 (9.26) 11.5 [8–17]	10.9 (8.74) 9 [6–13]	10 (8.59) 8 [2–7]	<0.001
SpO2 at admission (%)	90.18 (8.3) 92 [87.5–96]	90.33 (7.74) 92 [88–96]	89.17 (9.38) 91 [87–96]	91.03 (7.61) 94 [88–96]	0.12
Severity of illness at admission acc. to WHO	3.64 (0.64) 4 [3–4]	3.69 (0.63) 4 [3–4]	3.71 (0.6) 4 [3–4]	3.51 (0.68) 3 [3–4]	0.002
SARS-CoV-2 vaccination (≥1 dose)	161 (26.83%)	9 (4.5%) (*n* = 200)	65 (34.57%) (*n* = 188)	87 (52.1%) (*n* = 167)	<0.001
Comorbidities					
Cardiovascular diseases	375 (62.5%)	122 (61%)	110 (55%)	143 (71.5%)	0.003
Hypertension	329 (54.83%)	109 (54.5%)	96 (48%)	124 (62%)	0.019
Atrial fibrillation	79 (13.17%)	30 (15%)	20 (10%)	29 (14.5%)	0.265
Ischemic heart disease	109 (18.17%)	31 (15.5%)	35 (17.5%)	43 (21.5%)	0.285
Chronic circulatory failure	74 (12.33%)	23 (11.5%)	14 (7%)	37 (18.5%)	0.002
Diabetes	34 (22.33%)	48 (24%)	34 (17%)	52 (26%)	0.076
Respiratory diseases	59 (9.83%)	15 (7.5%)	14 (7%)	30 (15%)	0.011
Cancer history	94 (15.67%)	23 (11.5%)	23 (11.5%)	48 (24%)	<0.001
Chronic kidney disease	31 (5.16%)	10 (5%)	9 (4.5%)	12 (6%)	0.788
Obesity	86 (14.33%)	24 (12%)	36 (18%)	26 (13%)	0.142
Neurological comorbidities					
Past history of stroke/TIA	50 (8.33%)	27 (13.5%)	8 (4%)	15 (7.5%)	0.002
Dementia	47 (7.83%)	14 (7%)	10 (5%)	23 (11.5%)	0.046
Epilepsy	15 (2.5%)	6 (3%)	3 (1.5%)	6 (3%)	0.540
Parkinson’s disease	10 (1.67%)	3 (1.5%)	2 (1%)	5 (2.5%)	0.490
Multiple sclerosis	5 (0.83%)	2 (1%)	1 (0.5%)	2 (1%)	0.82
Neuromuscular disease	9 (1.5%)	4 (2%)	2 (1%)	3 (1.5%)	0.697
CNS Neoplasm	14 (2.33%)	7 (3.5%)	3 (1.5%)	4 (2%)	0.39
Systemic features					
Fever	389 (64.83%)	151 (75.5%)	129 (64.5%)	109 (54.5%)	<0.001
Cough	331 (55.17%)	119 (59.5%)	124 (62%)	88 (44%)	<0.001
Dyspnea	342 (57%)	141 (70.5%)	109 (54.5%)	92 (46%)	<0.001
Diarrhea	89 (14.83%)	34 (17%)	32 (16%)	23 (11.5%)	0.250
Runny nose	29 (4.83%)	3 (1.5%)	12 (6%)	14 (7%)	0.025
Sore throat	51 (8.5%)	14 (7%)	14 (7%)	23 (11.5%)	0.186
Rash	8 (1.33%)	1 (0.5%)	1 (0.5%)	6 (3%)	0.043
Weakness	361 (60.17%)	129 (64.5%)	117 (58.5%)	115 (57.5%)	0.269
Loss of appetite	120 (20%)	46 (23%)	43 (21.5%)	31 (15.5%)	0.134
Respiratory support					
No oxygen	142 (23.67%)	29 (14.5%)	40 (20%)	73 (36.5%)	<0.001
Low-dose oxygen therapy	453 (75.5%)	169 (84.5%)	157 (78.5%)	127 (63.5%)	<0.001
HFNOT	133 (21.17%)	45 (22.5%)	59 (29.5%)	29 (14.5%)	0.001
NIMV	45 (7.5%)	15 (7.5%)	14 (7%)	16 (8%)	0.930
IMV	48 (8%)	19 (9.5%)	15 (7.5%)	14 (7%)	0.622
Treatment					
Dexamethasone	403 (67.17%)	126 (63%)	166 (83%)	111 (55.5%)	<0.001
Chloroquine/hydroxychloroquine	16 (2.66%)	14 (7%)	1 (0.5%)	1 (0.5%)	<0.001
COVID-19 convalescent plasma	77 (12.83%)	76 (38%)	0 (0%)	1 (0.5%)	<0.001
Remdesivir	167 (27.83%)	42 (21%)	51 (25.5%)	74 (37%)	0.001
Tocilizumab	56 (9.33%)	9 (4.5%)	30 (15%)	17 (8.5%)	0.001
Baricitinib	20 (3.33%)	0 (0%)	10 (5%)	10 (5%)	0.006
Molnupiravir	12 (2%)	0 (0%)	0 (0%)	12 (6%)	<0.001
LMWH	543 (90.5%)	173 (86.5%)	188 (94%)	182 (91%)	0.036
Antibiotics	384 (64%)	173 (86.5%)	110 (55%)	101 (50.5%)	<0.001
Bacterial coinfections	122 (20.33%)	39 (19,5%)	26 (13%)	57 (18.5%)	<0.001
ICU admission	44 (7.33%)	14 (7%)	14 (7%)	16 (8%)	0.907
Death	113 (18.83%)	40 (20%) (*n* = 200)	35 (17.68%) (*n* = 198)	38 (19%) (*n* = 200)	0.838

Abbreviations: SpO2: oxygen peripheral blood saturation; WHO: World Health Organization; TIA: transient ischemic attack; CNS: central nervous system; HFNOT: high flow nasal oxygen therapy; NIMV: non-invasive mechanical ventilation; IMV: invasive mechanical ventilation; LMWH: low-molecular-weight heparin; ICU: intensive care unit.

**Table 2:** Laboratory results according to pandemic waves. Parameters are presented as median (interquartile range—IQR) (with Kruskal–Wallis test).

Variables	Patients (*n*)	Pre-Delta, Median (IQR)	Delta, Median (IQR)	Omicron, Median (IQR)	*p*-Value
CRP, mg/L	600	102.55 (39.34–170.91)	76.95 (34.38–134.2)	55.64 (15.13–139.94)	<0.001 pre-D>D, pre-D>O
D-dimer, ng/mL	585	1469 (802–3786)	1178 (656–3249)	1274 (618.5–3035)	0.12
Ferritin, ng/mL	444	850.38 (396.9–1561.7)	749.59 (358.1–1781.4)	471.05 (186.05–1106.59)	<0.001 pre-D>O, D>O
WBC, G/L	600	10.53 (6.97–14.7)	9.61 (5.5–14.61)	8.68 (4.28–12.45)	<0.001 pre-D>O
HGB, g/L	600	12.2 (10.7–13.5)	12.85 (11.55–14)	12.1 (10.6–13.4)	<0.001 D>pre-D, D>O
PLT, G/L	600	199 (145–389)	192.5 (146–277)	175 (128–239)	<0.001 pre-D>O, D>O
Procalcitonin, ng/mL	506	0.1 (0.05–0.54)	0.094 (0.04–0.25)	0.097 (0.04–0.52)	0.137
LDH, U/L	545	400 (306–556)	378 (284–554)	328 (249–451)	<0.001 pre-D>O, D>O
Interleukin-6 pg/mL	132	47.5 (24–125)	51.3 (10.5–137)	41.4 (17.1–99.2)	0.93

Abbreviations: CRP: C-reactive protein; WBC: white blood count; HGB: hemoglobulin; PLT: platelet count; LDH: lactate dehydrogenase; pre-D: pre-Delta; D: Delta; O: Omicron.

## Data Availability

All relevant data are within the manuscript.

## Supplementary Materials

The following supporting information can be downloaded at: https://www.mdpi.com/article/10.3390/jcm13123477/s1, Table S1: Comparison of neurological manifestations (symptoms and complications) in COVID-19 patients hospitalized across Pre-Delta, Delta and Omicron waves.
