# Supplementary material for: Complexity and Diversity of the Neurological Spectrum of SARS-CoV-2 over Three Waves of COVID-19
Source: J Clin Med. 2024 Jun 14;13(12):3477. doi: 10.3390/jcm13123477 (PMC11204600; doi:10.3390/jcm13123477)
Supplement: Supplementary file 1 [file jcm-13-03477-s001.zip › jcm-2983222-supplementary.pdf]

**Table S1.** Comparison of neurological manifestations (symptoms and complications) in COVID-19 patients hospitalized across Pre-Delta, Delta and Omicron waves (supplementary material).

| **Variables** | **Total patients (*n*= 600)** | | **Pre-Delta (*n* = 200)** | | **Delta (*n* =200)** | | **Omicron (*n* = 200)** | | **Chi² *P*-Value** |
|---|---|---|---|---|---|---|---|---|---|
| | **N** | % | **N** | % | N | % | N | % | |
| NEUROLOGICAL MANIFESTATIONS | 294 | 49 | 99 | 49.5 | 101 | 50.5 | 94 | 47 | 0.771 |
| **Neurological symptoms** | 289 | 48.17 | 95 | 47.5 | 100 | 50 | 94 | 47 | 0.813 |
| Headache | 68 | 11.33 | 23 | 11.5 | 23 | 11.3 | 22 | 11 | 0.984 |
| Dizziness | 34 | 5.67 | 10 | 5 | 7 | 3.5 | 17 | 8.5 | 0.852 |
| Myalgia | 68 | 11.33 | 23 | 11.5 | 29 | 14.5 | 16 | 8 | 0.122 |
| Smell disorder | 28 | 4.67 | 12 | 6 | 13 | 6.5 | 3 | 1.5 | 0.032 |
| Taste disorder | 32 | 5.33 | 12 | 6 | 17 | 8.5 | 3 | 1.5 | 0.007 |
| Vision disorder | 4 | 0.67 | 2 | 1 | 2 | 1 | 0 | 0 | 0.365 |
| Altered mentation | 90 | 15 | 36 | 18 | 24 | 12 | 30 | 15 | 0.244 |
| Mood disorder | 40 | 6.67 | 19 | 9.5 | 7 | 3.5 | 14 | 7 | 0.054 |
| Memory disorder | 28 | 4.67 | 5 | 2.5 | 14 | 7 | 9 | 4.5 | 0.102 |
| Sleep disorder | 21 | 3.5 | 8 | 4 | 3 | 1.5 | 10 | 5 | 0.149 |
| Paresthesia | 7 | 1.17 | 2 | 1 | 1 | 0.5 | 4 | 2 | 0.364 |
| Paresis | 20 | 3.33 | 10 | 5 | 2 | 1 | 8 | 4 | 0.069 |
| **Neurological complications** | 41 | 6.83 | 18 | 9 | 6 | 3 | 17 | 8.5 | 0.031 |
| Cerebrovascular diseases | 25 | 4.17 | 14 | 7 | 2 | 1 | 9 | 4.5 | 0.011 |
| TIA | 2 | 0.33 | 0 | 0 | 0 | 0 | 2 | 1 | 0.134 |
| Ischemic stroke | 18 | 3 | 12 | 6 | 2 | 1 | 4 | 2 | 0.008 |
| Hemorrhagic stroke | 2 | 0.33 | 1 | 0.5 | 0 | 0 | 1 | 0.5 | 0.605 |
| Venous thrombosis | 3 | 0.5 | 1 | 0.5 | 0 | 0 | 2 | 1 | 0.366 |
| Encephalopathy | 7 | 1.17 | 0 | 0 | 3 | 1.5 | 4 | 2 | 0.153 |
| Seizure | 6 | 1 | 2 | 1 | 1 | 0.5 | 3 | 1.5 | 0.603 |
| Ataxia | 1 | 0.17 | 0 | 0 | 0 | 0 | 1 | 0.5 | 0.367 |
| Myoclonus | 1 | 0.17 | 1 | 0.5 | 0 | 0 | 0 | 0 | 0.364 |
| Mononeuropathy | 1 | 0.17 | 0 | 0 | 0 | 0 | 1 | 0.5 | 0.368 |
| Polyneuropathy | 1 | 0.17 | 0 | 0 | 0 | 0 | 1 | 0.5 | 0.368 |
| GBS | 1 | 0.17 | 0 | 0 | 0 | 0 | 1 | 0.5 | 0.365 |
| Meningitidis | 3 | 0.5 | 1 | 0.5 | 0 | 0 | 2 | 1 | 0.367 |
| Encephalitis | 2 | 0.33 | 1 | 0.5 | 0 | 0 | 1 | 0.5 | 0.605 |

Abbreviations: TIA: transient ischemic attack, GBS: Guillain-Barré syndrome.
